# Outcomes of Surgical Patch Angioplasty of The Coronary Artery for Diffuse Coronary Artery Disease

**DOI:** 10.21470/1678-9741-2019-0390

**Published:** 2020

**Authors:** Dong Li, Pengfei Guo, Lei Chen, Yang Wu, Gang Wang, Cangsong Xiao

**Affiliations:** 1 Department of Cardiovascular Surgery, Medical School of Chinese PLA, Beijing, China.

**Keywords:** Coronary Artery Disease, Mammary Arteries, Cardiopulmonary Bypass, Retrospective Studies, Follow-Up Studies, Constriction, Coronary Artery Bypass, Angioplasty, Myocardial Revascularization, Prognosis

## Abstract

**Introduction:**

Diffuse coronary artery disease (CAD) has a poor prognosis and many patients are ineligible for conventional coronary artery bypass grafting (CABG). This study evaluated the 12-month outcomes of coronary artery reconstruction and surgical patch angioplasty of the coronary artery for diffuse CAD.

**Methods:**

A retrospective cohort study of patients who underwent CABG with surgical patch angioplasty of the coronary artery (reconstruction group) or standard CABG alone (standard group) at the Cardiovascular Surgery Department of the local Hospital between January 2014 and January 2016. Follow-up was censored at 12 months after surgery.

**Results:**

Cardiopulmonary bypass and aortic cross-clamping durations were longer in the reconstruction group (n=32) than in the standard group (n=125) (*P*<0.05). There were no differences in graft blood flow and postoperative levels of cardiac markers between the two groups (*P*>0.05). In the reconstruction group, one patient died; a vein graft showed occlusion. In the standard group, two patients died; one left internal mammary artery graft and three vein grafts showed occlusion. There were no significant differences in mortality, major adverse cardiovascular and cerebrovascular events, and patency between the two groups (*P*>0.05).

**Conclusion:**

Coronary artery reconstruction and surgical patch angioplasty of the coronary artery can be performed for diffuse CAD. Patient outcomes were not significantly different from those of patients who underwent standard CABG.

**Table:** 

Abbreviations, acronyms & symbols				
CABG	= Coronary artery bypass grafting		LAD	= Left anterior descending artery
CAD	= Coronary artery disease		LIMA	= Left internal mammary artery
CK-MB	= Creatine kinase MB		LMCA	= Left main coronary artery
CPB	= Cardiopulmonary bypass		MACCE	= Major adverse cardiovascular and cerebrovascular events
CTA	= Computed tomography angiography		MI	= Myocardial infarction
CT	= Computed tomography		PCI	= Percutaneous coronary intervention
GSV	= Great saphenous vein		SVG	= Saphenous vein graft
HTK	= Histidine-tryptophan-ketoglutarate		TTFM	= Transit-time flow measurement
ICU	= Intensive care unit			

## INTRODUCTION

Diffuse coronary artery disease (CAD) is generally considered in the presence of a narrowing ≥70%) of a coronary artery ≥20 mm or in the presence of multiple stenoses encompassing the whole length of the coronary artery^[1,2]^. Nevertheless, diffuse CAD can lead to severe myocardial ischemia and thereby angina, myocardial infarction (MI), and even heart failure, especially when the left anterior descending artery (LAD) is extensively involved^[1,3-5]^. The prognosis of diffuse CAD after stenting or coronary artery bypass grafting (CABG) is unknown.

One possible treatment for coronary artery stenosis is CABG, but many patients with diffuse CAD are ineligible to traditional CABG techniques^[1,6-10]^ or CABG does not achieve the desired results^[11,12]^. This is because the diffuse atherosclerotic lesions render the artery unsuitable for distal grafting^[13]^. 

A number of surgical techniques have been proposed^[13]^. Coronary arteries with diffuse stenosis can be effectively widened and reconstructed by CABG using patches taken from the left internal mammary artery (LIMA) or great saphenous vein (GSV) *in situ*, achieving good outcomes^[14]^. Coronary artery reconstruction and surgical patch angioplasty of the coronary artery have been described mainly for the treatment of isolated ostial stenosis of the left main coronary artery (LMCA), which is a rare condition^[15]^. This approach has also been described as a rescue surgery after percutaneous coronary intervention (PCI) complications^[16]^ and for the treatment of rare congenital heart conditions^[17]^. Nevertheless, considering the relative novelty of the technique and the low prevalence of diffuse CAD, data regarding the outcomes of this surgery for diffuse CAD are still lacking. 

Therefore, this study aimed to evaluate the outcomes of coronary artery reconstruction and surgical patch angioplasty of the coronary artery for diffuse CAD. 

## METHODS

### Study Design and Patients

This is a retrospective cohort study of patients who underwent coronary artery reconstruction and surgical patch angioplasty of the coronary artery (reconstruction group) or standard CABG alone (standard group) at the Cardiovascular Surgery Department of the Hospital between January 2014 and January 2016. 

The surgical indications were: 1) stable angina pectoris: conservative treatment was ineffective; coronary artery angiography showed that the coronary artery trunk or the stenosis of proximal end of anterior descending branch/circumflex artery was >70%, patients with lesions in three branches of the coronary artery, especially patients with low left ventricular ejection fraction of cardiac function; 2) unstable angina: typical angina that affects daily life and work, and conservative treatment was ineffective; coronary artery angiography showed that the coronary artery trunk or the stenosis of proximal end of anterior descending branch/circumflex artery was >70%; 3) severe coronary stenosis: three major branches of the coronary artery (anterior descending branch, circumflex artery, right coronary artery) had severe stenosis (>75%); and 4) failure of interventional treatment.

Patients who underwent CABG surgery by the same surgeon for CAD during the study period were included. Exclusion criteria were: 1) other cardiac surgery during CABG; 2) abnormal preoperative coagulation function or platelet count; or 3) preoperative cardiac dysfunction (left ventricular long diameter >60 mm or ejection fraction <50%). Data were extracted from the medical records.

This study was approved by the ethics committee of the local hospital. The need for individual consent was waived by the committee because of the retrospective nature of the study.

Surgical Procedures

Coronary artery reconstruction and surgical patch angioplasty of the coronary artery were performed in patients with diffuse stenosis of the target vessels. Conventional CABG was performed in patients with conventional non-diffuse stenosis.

All surgeries were performed under general anesthesia and cardiopulmonary bypass (CPB) by the same three surgeons, including a chief surgeon, an attending surgeon, and a surgeon. Sternotomy and establishment of CPB were routinely performed. 

**Fig. 1:**
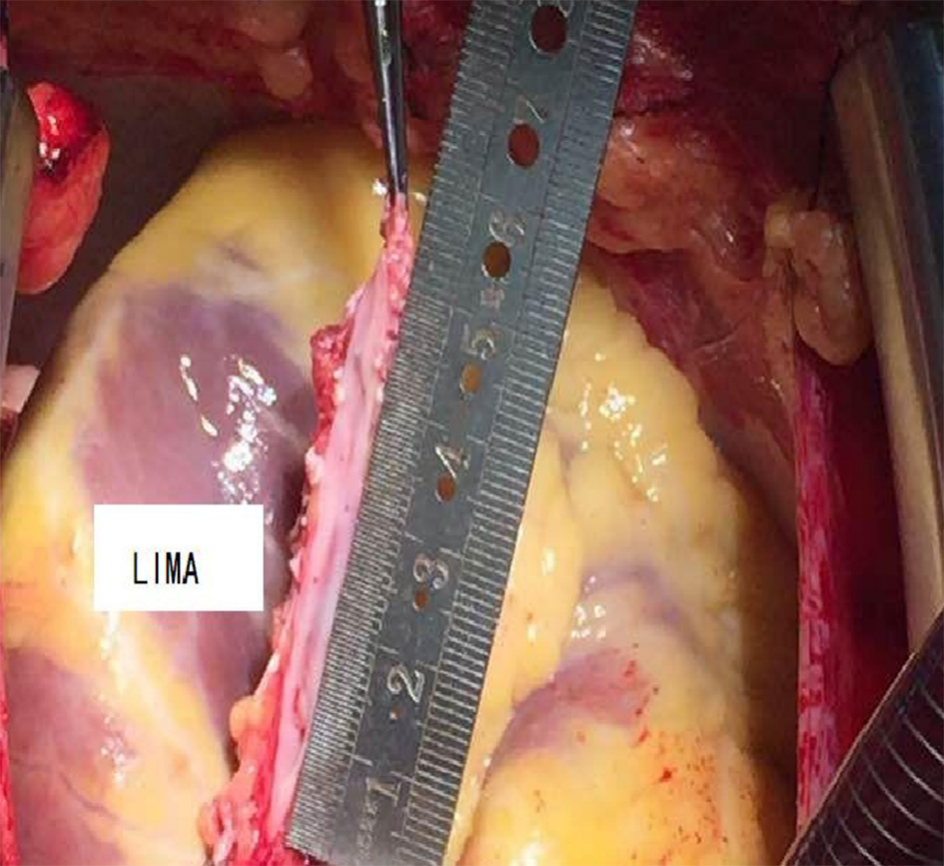
Onlay reconstruction and revascularization of the left anterior descending artery (LAD) with in situ pedicled left internal mammary artery (LIMA).

**Fig. 2:**
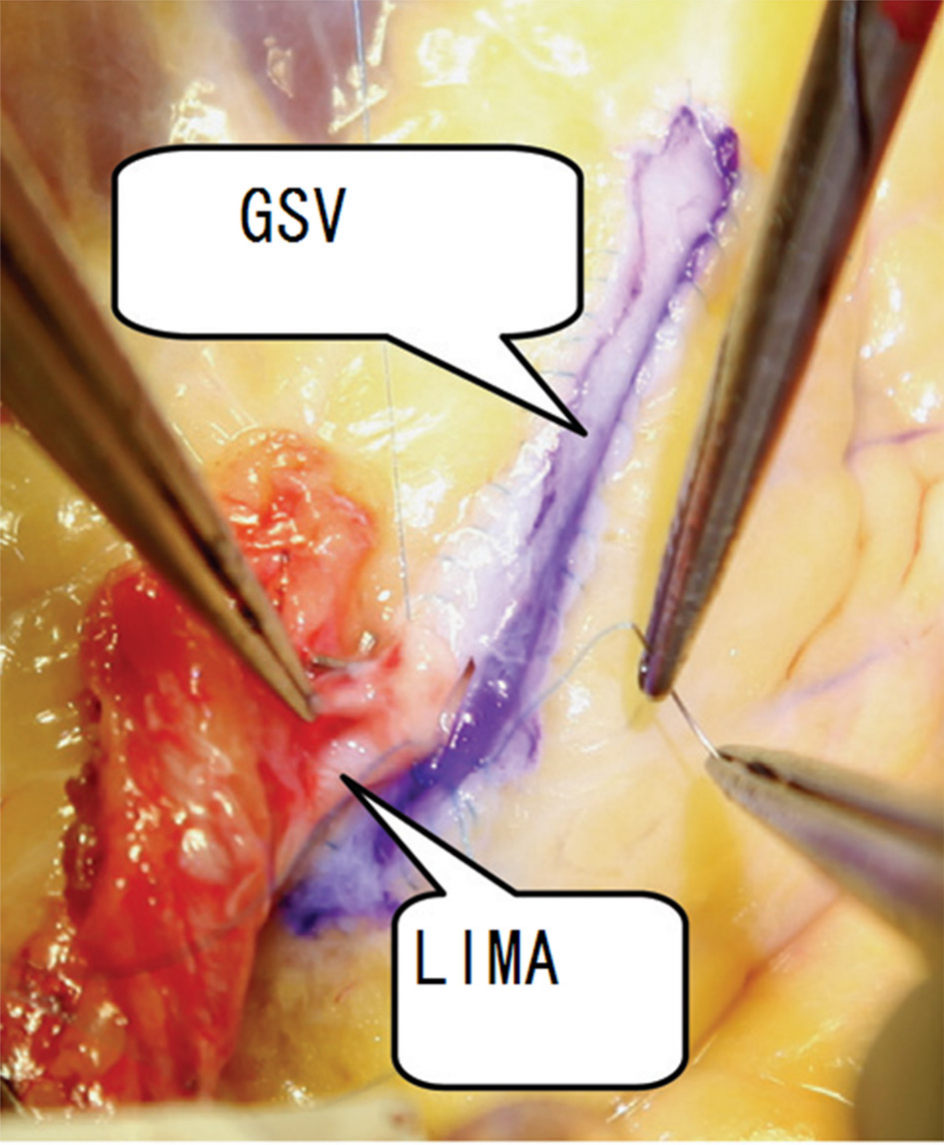
A great saphenous vein (SVG) was used for left anterior descending artery (LAD) onlay reconstruction. The left internal mammary artery (LIMA) was anastomosed to the GSV in an end-to-side fashion.

In the reconstruction group, patient-specific conditions were determined by angiography performed by experienced technicians (Innova 3100-IQ, GE Healthcare, Waukesha, WI, USA; contrast: Optiray 320, Mallinckrodt Pharmaceuticals, Staines-upon-Thames, UK). Then, surgery was performed according to the angiography results: 1) an anastomosis was established between the LIMA patch and LAD; 2) LAD was reconstructed with a saphenous vein graft (SVG) patch and anastomosis of LIMA to the SVG patch; or 3) target arteries with diffuse disease, except for LAD, were reconstructed using SVG. In each patient, the incision started from the middle portion of the LAD and continued towards its distal and proximal ends. At the distal end of LAD, the incision was terminated in a proper position in the coronary artery lumen; at the proximal end, a major stenosis was left intact in order to avoid competitive flow in the postoperative period. For each of the patients undergoing *in situ* LIMA patch reconstruction, the distal ventral portion of LIMA was cut longitudinally to obtain an arterial patch whose length fit the incision made in LAD. Then the patch was continuously sutured to LAD using 7/0 Prolene suture for reconstruction (Figure 1). For each of the patients undergoing SVG reconstruction, SVG was incised longitudinally to obtain an SVG with proper length and width and then SVG was used to reconstruct the LAD. An end-to-side anastomosis was created between LIMA and SVG (Figure 2). The suturing ensured that atherosclerotic plaques were isolated from the coronary artery lumen after angioplasty.

For the patients who underwent standard CABG, LIMA was anastomosed to LAD, and SVG was anastomosed with other diseased target arteries. 

For patients with diffuse disease of coronary arteries except LAD, the distal stenoses to be bypassed were cut, with the incisions extending to normal positions in arterial walls at both the proximal and distal ends (both incisions’ ends were usually about 2 mm beyond the boundaries of the stenoses). The incision in each SVG was prolonged to fit the length of the target artery. SVG and target artery were continuously anastomosed by end-to-side anastomosis using 7/0 Prolene suture. After angioplasty, the atherosclerotic plaques were separated from the lumen of the arteries. 

All patients included in this study did not undergo coronary endarterectomy. After the end of CPB, a transit-time flow measurement (TTFM) was used to measure and record blood flow in the grafts and ensure it was satisfactory before closing the chest. 

The patients routinely received 75 mg clopidogrel and 100 mg oral enteric-coated aspirin immediately after tracheal tube removal on the morning of the 1^st^ operative day, for 1 year. CK-MB and troponin T (COADS-8000, Roche, Germany) levels in peripheral blood were routinely monitored on postoperative days 1-3.

### Follow-Up

Patients were routinely followed at 6 and 12 months, and then annually. Coronary artery CT angiography was performed using 64-MSCT (SOMATOM Sensation Cardiac 64, Siemens, Erlangen, Germany) to verify graft patency at 6 months. Mortality, major adverse cardiovascular and cerebrovascular events (MACCE) (including death, non-fatal myocardial infarction, revascularization, stroke, and cerebral hemorrhage), graft patency, and complications were extracted from medical records.

### Statistical Analysis

Statistical analysis was performed using SPSS 19.0 (IBM, Armonk, NY, USA). Continuous data are presented as mean ± standard deviation and were analyzed using the Student’s t-test. Categorical data are presented as percentage and were analyzed by chi-square test or Fisher’s exact test, as appropriate. Two-sided *P*-values <0.05 were considered statistically significant.

## RESULTS

### Patient Characteristics

During the study period, 32 patients (62.1±9.4 years; 71.9% male; 23.5±3.8 kg/m^2^) underwent coronary artery reconstruction and surgical patch angioplasty of the coronary artery (reconstruction group) and 128 patients (63.7±11.4 years; 83.6% male; 23.2±3.6 kg/m^2^) underwent CABG alone (standard group). Table 1 presents the preoperative data of the patients. There were no significant differences between the two groups in terms of age, gender, angina, number of diseased arteries, left ventricular systolic function, myocardial infarction (MI), hypertension, diabetes, cerebrovascular diseases, and chronic renal insufficiency (all *P*>0.05).

**Table 1:** Comparison of preoperative data.

	Reconstruction (n=32)	Standard (n=128)	*P*-value
Age (years)	62.1±9.4	63.7±11.4	0.83
Gender, male, n (%)	23 (71.9)	107 (83.6)	0.21
BMI (kg/m^2^)	23.5±3.8	23.2±3.6	0.66
Smoking history, n (%)	19 (59.4)	73 (57.0)	0.84
MI, n (%)	9 (28.1)	36 (28.1)	0.92
Left ventricular ejection fraction (%)	59.5±11.5	62.1±9.9	0.48
Number of diseased arteries, n (%)			
2	3 (9.4)	13 (10.2)	
3	29 (90.6)	115 (89.8)	
Diabetes, n (%)	10 (31.3)	48 (37.5)	0.76
Hypertension, n (%)	18 (26.3)	75 (58.6)	0.68
Peripheral vascular diseases, n (%)	3 (9.4)	5 (3.9)	0.32
Cerebrovascular diseases, n (%)	4 (12.5)	15 (11.7)	0.91
Chronic renal insufficiency, n (%)	1 (3.1)	3 (3.9)	>0.99

BMI=body mass index; MI=myocardial infarction

### Intraoperative and Postoperative Data

In the reconstruction group, the bypasses were 3.5±0.9 cm in length. An anastomosis was established between the LIMA patch and LAD in 18 cases, and 4 cases underwent LAD reconstruction with SVG patch and anastomosis of LIMA to SVG patch. The 10 patients with target arteries other than LAD underwent reconstruction using the SVG surgical technique; there were one case of stenotic diagonal branch artery, five of diseased posterior descending artery, two of diseased posterior branch of left ventricle, and two of stenotic obtuse marginal branch. 

After surgery, patients had no angina and were discharged from the hospital after recovery. Complications such as perioperative MI, low cardiac output, malignant ventricular arrhythmias, reoperation for hemostasis, stroke and renal insufficiency did not occur. Table 2 shows the intraoperative and postoperative data of both groups. The proportion of patients with two-vessel disease was higher in the reconstruction group than in the standard group (31.3% *vs*. 10.9%, *P*=0.01). There were no significant differences in blood flow (LAD and SVG), duration of ventilator use, intensive care unit (ICU) stay, postoperative atrial fibrillation, postoperative MI, postoperative renal failure, postoperative stroke, ventricular arrhythmias, postoperative mortality rate and complications between the two groups (both *P*>0.05). The duration of CPB (116.5±21.3 *vs*. 97.2±29.6 min, *P*=0.04) and aortic cross-clamping (95.3±21.5 *vs*. 66.9±20.7 min, *P*<0.01) in the reconstruction group were significantly longer than in the standard group. There were no significant differences in CK-MB and troponin T levels on 3^rd^ postoperative day (all *P*>0.05) (Table 3).

**Table 2:** Intraoperative and postoperative data.

	Reconstruction (n=32)	Standard (n=128)	*P*-value
Number of bypassed arteries, n (%)			0.01
2	10 (31.3)	14 (10.9)	
3	14 (43.8)	76 (59.4)	
4	8 (25.0)	38 (29.7)	
Blood flow in LAD (ml/min)	35.3±8.7	28±13.6	0.51
Blood flow in SVG (ml/min)	39.1±18.6	36.1±14.8	0.96
Cardiopulmonary bypass time (min)	116.5±21.3	97.2±29.6	0.04
Aortic cross-clamping time (min)	95.3±21.5	66.9±20.7	<0.01
Ventilator support time (h)	18.8±9.1	18.7±11.3	0.74
ICU length of stay (d)	3.1±1.5	3.3±1.3	0.09
Postoperative atrial fibrillation, n (%)	3 (9.4)	15 (11.7)	0.68
Postoperative MI, n (%)	0	2 (1.6)	0.75
Postoperative renal insufficiency, n (%)	1 (3.1)	2 (1.6)	0.79
Postoperative stroke, n (%)	0	2 (1.6)	0.88
Ventricular arrhythmias, n (%)	0	1 (0.8)	0.93

ICU=intensive care unit; LAD=left anterior descending artery; MI=myocardial infarction; SVG=saphenous vein graft

**Table 3:** Postoperative levels of myocardial enzymes.

	Reconstruction (n=32)	Standard (n=128)	*P*-value
Troponin T (ng/ml)			
1^st^ postoperative day	0.3±0.1	0.3±0.2	0.58
2^nd^ postoperative day	0.3±0.2	0.3±0.3	0.89
3^rd^ postoperative day	0.2±0.1	0.3±0.1	0.67
CK-MB (ng/ml)			
1^st^ postoperative day	12.0±5.4	11.3±6.4	0.59
2^nd^ postoperative day	7.0±0.8	7.7±0.5	0.73
3^rd^ postoperative day	2.4±0.8	3.3±1.7	0.55

CK-MB=creatine kinase MB

### Follow-Up

In the reconstruction group, all 32 patients were followed (100%). During follow-up, one patient died from cerebral hemorrhage. All 31 remaining patients underwent coronary computed tomography angiography (CTA) follow-up. All LIMA grafts were patent, and one vein graft showed occlusion. In the standard group, 125 patients were followed (98%). Two patients died, one from heart failure combined with pulmonary infection and the other from recurrent MI. Coronary CTA follow-up was performed in 120 patients. One LIMA graft and three vein grafts showed occlusion. There were no significant differences in mortality rate, occurrence of MACCE (death, non-fatal myocardial infarction, revascularization, stroke, and cerebral hemorrhage), and patency rate between the two groups (Table 4).

**Table 4:** Follow-up.

	Reconstruction (n=32)	Standard (n=125)	*P*-value
Mortality rate, n (%)	1 (3.1)	2 (1.6)	0.87
Incidence of MACCE, n (%)	2 (6.3)	7 (5.6)	0.77
LIMA graft patency rate, n (%)	32 (100.0)	119 (99.2)	0.48
Vein graft patency rate, n (%)	31 (96.9)*	118 (98.3)**	0.56

LIMA=left internal mammary artery; MACCE=major adverse cardiovascular and cerebrovascular events

* A patient can undergo bypass with multiple venous bridges; 32 patients had a total of 62 venous bridges; one failed; the patency rate was 61/62 (98.4%).

** A patient can undergo bypass with multiple venous bridges; 120 patients had a total of 264 venous bridges; three failed; the patency rate was 261/264 (98.7%).

## DISCUSSION

This study suggests that coronary artery reconstruction and surgical patch angioplasty of the coronary artery can be performed for diffuse CAD. Patient outcomes were not significantly different from those of patients who underwent standard CABG.

Complete revascularization is commonly accepted in CABG. If a coronary artery has a long segment of stenosis, simple CABG is not effective in unblocking all diseased vessels. For patients with diffuse coronary artery stenosis, incomplete revascularization is an independent risk factor that influences prognosis^[1,18]^. For patients with diffuse disease in LAD and its interventricular branch, in particular, revascularization cannot achieve the desired results and is considered as a major risk factor for postoperative MACCE^[19]^. Diffuse coronary artery stenosis can not only complicate surgical procedures, but also reduce long-term graft patency^[20]^. Nevertheless, patients with diffuse CAD will still have a worse prognosis if they do not receive CABG, despite the difficulty and high risk in performing CABG on them^[1]^. A follow-up survey of patients who were denied CABG because of diffuse CAD found that 39.2% of these patients died of cardiac causes, 37.2% died of acute MI, and 5.8% developed congestive heart failure^[21]^.

Coronary artery reconstruction is one of the effective methods for treating severe diffuse CAD^[22]^. Through a comparison of coronary artery reconstruction with endarterectomy, Fukui et al.^[11]^ found that a higher patency rate can be achieved using LIMA patch reconstruction. A 3-year follow-up survey showed that the patency rate of artery in the reconstruction group reached 97.6%, compared to 89.7% in the endarterectomy group. A study by Ogus et al.^[23]^ reported good short- and long-term results of LIMA patch reconstruction; the mortality rate in the early postoperative period was 1.9%; the 3-year, 5-year, and 7-year survival rates were 93.8%, 89.6%, and 85.5%, respectively; and the corresponding rates of angina relief were 94.5%, 88.5%, and 82.9%, respectively. In the present study, no perioperative death occurred, angina was relieved in all patients, and no case exhibited any major complication. Despite the small number of patients in the reconstruction group, these results strongly suggest, to some extent, that this method could be safe and effective to achieve complete revascularization, but prospective trials are necessary to reach firm conclusions.

Reconstruction of LAD with diffuse stenosis can be achieved by two methods. The first method is to widen and reconstruct LAD using a LIMA patch. The second method is to widen and reconstruct the target artery with a GSV patch and then establish an end-to-side anastomosis between the LIMA graft and the venous patch. The former is more frequently adopted and can deliver satisfactory results^[11]^. In the reconstruction group, 18 patients underwent LAD reconstruction with LIMA patch and four with SVG patch. The arteries were patent during the follow-up period. There are three technical points that are essential for widening the target arteries. First, incisions in the target arteries should extend beyond the boundaries of the stenoses and the anastomotic stomas should reach normal coronary arteries. This could, in theory, substantially decrease the incidence of intimal hyperplasia. The second point is to make sure that the collateral vessel openings are located within the lumen during suture, which can effectively improve target artery perfusion. The third point is to isolate the atherosclerotic plaques from the lumen after angioplasty. As the rupture of atherosclerotic plaques with thin and weak fibrous caps is the major cause of thrombosis, unstable angina and MI, keeping these atherosclerotic plaques out of the reconstructed coronary artery lumen can lower the rates of perioperative MI and postoperative long-term cardiac events.

The method of coronary artery reconstruction and surgical patch angioplasty of the coronary artery for treatment of diffuse CAD requires a long duration of surgery, leading to prolonged periods of CPB and aortic cross-clamping. Existing myocardial protection techniques can meet the requirements of complex and long surgical procedures and will not increase the risk of surgery. One possibility is the use of one-off infusion of HTK solution, which can provide protection for over 2 hours^[24]^. This is longer than the duration of myocardial ischemia of patients in the reconstruction group, at 73±21 minutes. During operation, the low temperature and cooling of the heart surface can lengthen to some extent the safe duration of myocardial ischemia. Due to the good results of reconstruction, satisfactory blood flows in grafts were achieved, thus lowering the risks of postoperative MI and low cardiac output^[25]^ and increasing the patency rates of the arteries. Among the patients in the reconstruction group, perioperative MI and low cardiac output were not observed, and the short- to medium-term patency rates were satisfactory. 

The present study has limitations. The main limitation of the study is that it compared two different surgeries performed for two different indications/diseases. This is because it would be unethical to perform standard CABG in patients with diffuse CAD. In addition, the sample size was small, and all patients were from a single center. In addition, the follow-up was short.

## CONCLUSION

In conclusion, for patients with long-segment or multiple diffuse coronary artery stenoses, performing coronary artery reconstruction and bypass grafting with LIMA or SVG patch through incisions on stenoses does not seem to pose additional surgical risk and achieved short-term results that could be comparable to standard CABG performed in patients with "regular" (non-diffuse) CAD. Given the novelty and immaturity of this technique, further research is needed to investigate the long-term graft patency rate, incidence of cardiac events, and other relevant issues.

**Table:** 

Authors' roles & responsibilities	
DL	Substantial contributions to the conception or design of the work; or the acquisition, analysis or interpretation of data for the work; drafting the work or revising it critically for important intellectual content; final approval of the version to be published
PG	Substantial contributions to the conception or design of the work; or the acquisition, analysis or interpretation of data for the work; final approval of the version to be published
LC	Substantial contributions to the conception or design of the work; or the acquisition, analysis or interpretation of data for the work; final approval of the version to be published
YW	Substantial contributions to the conception or design of the work; or the acquisition, analysis or interpretation of data for the work; final approval of the version to be published
GW	Substantial contributions to the conception or design of the work; or the acquisition, analysis or interpretation of data for the work; final approval of the version to be published
CX	Substantial contributions to the conception or design of the work; or the acquisition, analysis or interpretation of data for the work; drafting the work or revising it critically for important intellectual content; final approval of the version to be published
